# Resting-State Electroencephalography Functional Connectivity Networks Relate to Pre- and Postoperative Language Functioning in Low-Grade Glioma and Meningioma Patients

**DOI:** 10.3389/fnins.2021.785969

**Published:** 2021-12-08

**Authors:** Nienke Wolthuis, Djaina Satoer, Wencke Veenstra, Marion Smits, Michiel Wagemakers, Arnaud Vincent, Roelien Bastiaanse, Perumpillichira J. Cherian, Ingeborg Bosma

**Affiliations:** ^1^Center for Language and Cognition Groningen, University of Groningen, Groningen, Netherlands; ^2^Department of Neurosurgery, Erasmus MC – University Medical Center Rotterdam, Rotterdam, Netherlands; ^3^Department of Rehabilitation Medicine, University Medical Center Groningen, University of Groningen, Groningen, Netherlands; ^4^Department of Radiology & Nuclear Medicine, Erasmus MC – University Medical Center Rotterdam, Rotterdam, Netherlands; ^5^Brain Tumour Centre, Erasmus MC Cancer Institute, Rotterdam, Netherlands; ^6^Department of Neurosurgery, University Medical Center Groningen, Groningen, Netherlands; ^7^National Research University Higher School of Economics, Moscow, Russia; ^8^Department of Neurology, University Medical Center Rotterdam, Rotterdam, Netherlands; ^9^Division of Neurology, Department of Medicine, McMaster University and Hamilton Health Sciences, Hamilton, ON, Canada; ^10^Department of Neurology, University Medical Center Groningen, Groningen, Netherlands

**Keywords:** language, network, functional connectivity, low-grade glioma, meningioma

## Abstract

**Introduction:** Preservation of language functioning in patients undergoing brain tumor surgery is essential because language impairments negatively impact the quality of life. Brain tumor patients have alterations in functional connectivity (FC), the extent to which brain areas functionally interact. We studied FC networks in relation to language functioning in glioma and meningioma patients.

**Method:** Patients with a low-grade glioma (*N* = 15) or meningioma (*N* = 10) infiltrating into/pressing on the language-dominant hemisphere underwent extensive language testing before and 1 year after surgery. Resting-state EEG was registered preoperatively, postoperatively (glioma patients only), and once in healthy individuals. After analyzing FC in theta and alpha frequency bands, weighted networks and Minimum Spanning Trees were quantified by various network measures.

**Results:** Pre-operative FC network characteristics did not differ between glioma patients and healthy individuals. However, hub presence and higher local and global FC are associated with poorer language functioning before surgery in glioma patients and predict worse language performance at 1 year after surgery. For meningioma patients, a greater small worldness was related to worse language performance and hub presence; better average clustering and global integration were predictive of worse outcome on language function 1 year after surgery. The average eccentricity, diameter and tree hierarchy seem to be the network metrics with the more pronounced relation to language performance.

**Discussion:** In this exploratory study, we demonstrated that preoperative FC networks are informative for pre- and postoperative language functioning in glioma patients and to a lesser extent in meningioma patients.

## Introduction

Primary brain tumors in the language dominant hemisphere are estimated to cause language impairment in nearly half of all patients (Davie et al., 2009). Language abilities are essential for everyday communication and for participation in society, hence, impairments negatively affect the quality of life (Hilari et al., 2012). As language covers a range of functions, several language modalities (speech production, comprehension, reading, and writing) and linguistic levels (e.g., phonology, concerning speech sounds; semantics, concerning meaning; and grammar, concerning word and sentence structure) are at risk in brain tumor patients.

The most frequently occurring primary brain tumors are gliomas and meningiomas. Gliomas are infiltrative tumors whereas the meningioma are extra-axially located Patients with a low-grade glioma involving eloquent brain areas generally undergo surgical resection by an awake procedure, to maximize the extent of resection while preventing additional impairment by surgical injury to eloquent brain areas. Patients of both groups have a relatively long life expectancy after surgery (Van Alkemade et al., 2012; Ho et al., 2014). Preservation of language functioning is crucial in these patients for successfully carrying out daily activities and maintaining work or continuing education. The incidence of language impairments after brain tumor surgery ranges between 10 and 70% (Santini et al., 2012; Antonsson et al., 2018; Di Cristofori et al., 2018; Van Kessel et al., 2020). Hence, it is important to gain more insight into the underlying mechanisms of language impairments in this group of patients, to identify at-risk patients and potentially prevent postoperative language decline.

Optimal functioning of the brain requires interactions between and within brain regions leading to a complex network organization. The properties of these interactions can be studied by using for instance diffusion tract imaging as a structural, and electroencephalography (EEG), magnetoencephalography (MEG) or functional magnetic resonance imaging (fMRI) as functional imaging techniques. Functional connectivity (FC) concerns the extent to which brain areas functionally interact, which is reflected by the level of synchronization of brain activity between different brain regions, characterized by any statistical interdependency between two signals (Aertsen et al., 1989).^1^ Intracranial tumors can, due to the location, growth and treatment, lead to alterations in these functional interactions using EEG and MEG and therefore adjust their organization in the brain (network organization).^2^ In brain tumor patients before surgery, FC tends to be increased in lower frequency bands (delta and theta) and decreased in higher frequency bands (beta and gamma) compared to healthy individuals using (MEG) (Bartolomei et al., 2006a,b; Van Dellen et al., 2012; Hu et al., 2013). For the (intermediate) alpha band, FC in brain tumor patients may be higher or lower than in a healthy population (Bartolomei et al., 2006a; Bosma et al., 2008), but appears to increase between pre- and postoperative assessments (Van Dellen et al., 2013; Lizarazu et al., 2020).

Network organization in brain tumor patients, as investigated with resting-state MEG, has been shown to relate to poorer cognitive functioning, such as verbal memory, working memory, attention, and executive functioning (Bosma et al., 2008, 2009; Van Dellen et al., 2012, 2013; Hu et al., 2013; Van Nieuwenhuizen et al., 2018; Derks et al., 2019). However, literature on language functioning in relation to FC network characteristics using EEG and MEG is scarce. Resting state fMRI on the other hand is a technique, believed to depend on the neurovascular uncoupling causing changes in neurometabolites. Language network studies done using resting state (rs) fMRI have shown differences in the network organization before and after surgery in glioma patients (Deverdun et al., 2020), and also, there is literature on the extent of the network involved in language function even in the contralateral hemisphere although impacting the core network to a lesser extent (Jin et al., 2021). MEG studies has reported that FC network alterations in the theta, alpha, and gamma bands are associated with poorer performance on a verbal fluency test in glioma patients (Van Dellen et al., 2012; Hu et al., 2013; Derks et al., 2019).^3^ Furthermore, Lizarazu et al. (2020) assessed left-hemispheric, low-grade glioma patients with an object naming test and semi-spontaneous speech ratings, and registered resting-state MEG before, as well as at 3 and 6 months after surgery. No relations between FC change in the alpha band and language change scores were found, possibly because language performance did not significantly change between the time points. Nonetheless, studies in patients with non-tumor-related brain injury show that FC network characteristics predict the type of language impairment and the course of language recovery (Castellanos et al., 2010; Nicolo et al., 2015; Ranasinghe et al., 2017).

The aim of the study is to ascertain differences in network organization of patients with a presumed low-grade glioma and meningioma patients preoperatively, compared to healthy participants and the relation to language function. Our hypothesis is that network metrics are predictive of postoperative language performance in glioma patients.

## Materials and Methods

### Participants

Patients with a presumed low-grade glioma and patients with a presumed grade I meningioma, who were planned to undergo surgery at the University Medical Centre Groningen (UMCG) or the Erasmus MC University Medical Centre Rotterdam (Erasmus MC), were invited to participate in the study and inclusion took place in 2017 and 2018.

Inclusion criteria were:

• intracranial, supratentorial, untreated tumor infiltrating into or pressing on the language-dominant hemisphere^4^ (if language lateralisation was unknown: right-handed patients with left-sided tumor);
• tumor diameter > 3 cm (meningiomas only);
• age between 18 and 75 years;
• in case of epilepsy, seizures well-controlled with anticonvulsants.

Exclusion criteria were:

• non-native speaker of Dutch;
• history of a medical, neurological or psychiatric condition known to affect language or cognitive functioning;
• history of substance abuse;
• consistent use of dexamethasone preoperatively;
• previous brain surgery or cranial radiation therapy.

Glioma patients underwent tumor resection with an awake procedure and meningioma patients underwent surgery under general anesthesia. Healthy volunteers were invited to participate in the control group and matched for age, gender and education (often proxies of the patients). The same exclusion criteria applied for this group. This multicentre study was approved by the medical, ethical review board of the UMCG, which was also applicable to the Erasmus MC. All participants gave written informed consent.

Fifteen glioma and 15 healthy participants and 10 meningioma with 9 healthy participants were included in the study. Their specifics are given in the “Result” section.

### Procedure

Both patient groups underwent extensive language assessment at two time points: T1 = before surgery (glioma patients: mean 33 days, range 6–83; meningioma patients: mean 20 days, range 1–48); and T2 = 12 months after surgery (glioma patients: mean 12.61, range 11.05–15.22; meningioma patients: mean 11.97, range 11.21–12.49). EEG registration was performed at T1 and at T2 in glioma patients, at T1 in meningioma patients, and once in healthy participants.

### Language Assessment

A selection of standardized language tests was conducted, assessing a wide range of linguistic abilities. Tests included object naming (De Witte et al., 2015), action naming in sentence context (Rofes, 2012), the shortened version of the Token Test (De Renzi and Faglioni, 1978), category fluency (Luteijn and Barelds, 2004), letter fluency (Schmand et al., 2008), reading and writing (Van Ierschot, 2018), and subtests of the Diagnostic Instrument for Mild Aphasia (DIMA; Satoer et al., 2019): repetition, semantic odd-picture-out, sentence completion), and sentence judgment (De Witte et al., 2015).

Raw scores were normalized by conversion to *z*-scores, using pre-existing normative data from a healthy population. Subsequently, eight language domains were created for the purpose of this study, as shown in Table 1. This was done for two reasons: (1) to reduce the number of statistical comparisons; and (2) to examine all language modalities (speech production, comprehension, reading, and writing), three linguistic levels (phonology, semantics, and grammar), and comprehension of two types of input (auditory and visual). A domain score equalled the average *z*-score of the tests in that domain for each patient. Domain scores below –1.5 indicated an impairment.

**TABLE 1:** Language domains under investigation with their corresponding tests.

Domains	Tests
(1) Production-Word retrieval	Object naming
(2) Production-Phonology	Repetition
	Letter fluency
(3) Production-Semantics	Semantic odd-picture-out
	Category fluency
(4) Production-Grammar	Sentence completion
	Action naming in sentence context
(5) Comprehension-Auditory input	Token Test
(6) Comprehension-Visual input	Sentence judgment (accuracy)
(7) Reading	Reading
(8) Writing	Writing

### Electroencephalography Registration

**FIGURE 1:**
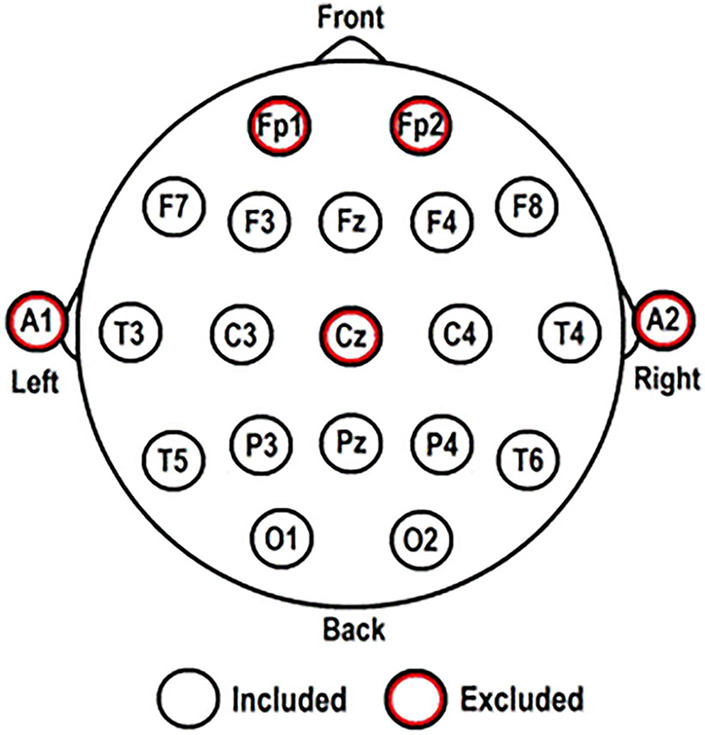
Electrode positions according to the International 10-20 System. The five red-circled electrodes were excluded from all analyses.

Electroencephalography was registered using 44-channel Schwarzer amplifiers (Natus Europe GmbH, Munich). Twenty-one scalp electrodes were applied according to the International 10-20 System, with or without caps according to local protocol. Electrode positions are shown in Figure 1. Additional polygraphic electrodes were applied for artifact detection: electrooculography (EOG; two diagonally placed electrodes for eye movements), electrocardiography (ECG; chest electrode), and respiration (abdominal movements). EEG was recorded with impedances ≤ 5 kΩ, a sampling frequency of 500 Hz, and with Cz as reference electrode.

At least 5 min of eyes-closed, resting-state EEG were registered for every participant. If there were many artifacts, the duration was prolonged to 10 min. Participants were instructed to refrain from moving and to stay alert. Alertness was continuously monitored and acoustic stimuli were given when signs of drowsiness appeared. For (glioma) patients with history of seizures and suspected epileptiform activity, the resting-state registration was followed by the standard clinical EEG registration of at least 30 min.

### Electroencephalography Processing

Artifact-free EEG for analysis was selected by visual inspection (IB) and segmented into five epochs of 8.19 s (4096 samples) for each participant. Using BrainVision Analyzer 2.0 (Brain Products GmbH, 2013). EEG data were re-referenced to a new averaged reference consisting of 16 scalp electrodes: F3; F4; F7; F8; T3; T4; T5; T6; C3; C4; P3; P4; O1; O2; Fz; and Pz (excluding: Cz [reference electrode]; Fp1 and Fp2 [to minimize excess muscle activity and eye movement artifacts]; A1 and A2 [do not register brain activity]). Only these 16 electrodes were included for FC network analysis, described below (for more detailed explanations on FC network analysis see Van Straaten and Stam, 2013; Zhang et al., 2014; Van Diessen et al., 2015).

### Functional Connectivity Network Analysis

Functional connectivity network analysis was performed in BrainWave software (version 0.9.152.12.26; Stam, 2018) for every EEG segment. The mean values of five segments for each participant were used in further (statistical) analyses. FC networks were analyzed in the theta (4–8 Hz) and alpha (8–13 Hz) frequency bands. These bands were selected based on previous literature (Van Dellen et al., 2012; Derks et al., 2019).

Functional connectivity network analysis was performed with the phase lag index (PLI), a correlation coefficient that measures synchronization of brain activity between different brain regions, by the consistency with which one signal is leading or lagging in phase with respect to another signal (Stam et al., 2007). As this is a phase-based technique, it does not depend on the amplitude of the signal. Other advantages of the PLI are that it analyses non-linear relations and that it is relatively insensitive to the effects of volume conduction, a phenomenon denoting that the activity recorded over different brain regions can be influenced by one single underlying source (Stam et al., 2007).

Analysis with the PLI results in values between 0 and 1 for every pair of signals. A PLI close to 0 demonstrates no/very weak synchronization between two signals recorded from different electrode sites, which indicates no/very weak functional interaction between the underlying brain areas. A PLI close to 1 indicates strong synchronization between two signals and a strong functional interaction between the underlying brain areas. PLI values form the basis for the FC networks, for both weighted and Minimum Spanning Tree (MST) networks, which provide complementary information. The organization of FC networks was quantified by network measures. Scores on these measures were referred to as network characteristics.

### Graph Theory and Network Measures

All network measures used are based on graph theory (Watts and Strogatz, 1998). This is a mathematical approach by which a complex network is reduced to a ‘graph,’ an abstract representation of the network. A graph consists of nodes and connections, which are links between nodes. In our case, nodes refer to the electrode sites, which represent the underlying brain areas, and connections represent functional interactions between the underlying brain areas.

Different aspects of the network organization were evaluated by using network measures. First, connectivity of individual nodes was examined. Nodes with many connections and a central position in the network are called ‘hubs.’ Subsequently, short-distance or local FC, and long-distance or global FC were examined. Lastly, efficiency of the entire network configuration was examined.

**FIGURE 2:**
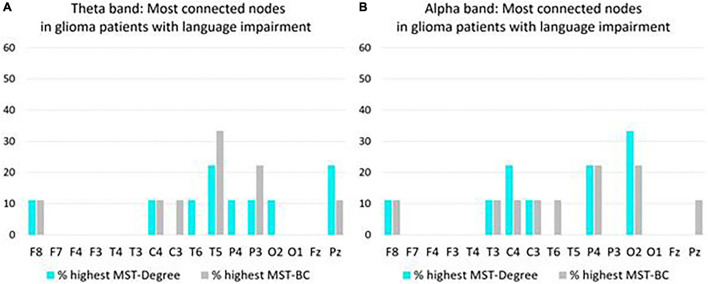
Overview of most connected nodes in MST networks in the theta band **(A)** and in the alpha band **(B)** in preoperative glioma patients with language impairment (*N* = 9). For each node, the *y*-axis expresses the percentage of patients who have the highest Degree/Betweenness Centrality (BC) ranking for that node in their MST network. If a patient’s network has two or more nodes with a shared highest ranking, all shared highest rankings are included in the figure.

Table 2 gives an overview of the network measures used, including examples corresponding to Figure 2. The first part of the table quantifies the original weighted networks, and the latter part quantifies the MST networks.

**TABLE 2:** Network measures used for the quantification of the functional connectivity (FC) brain networks.

Measure		Evaluation of	Description	Interpretation
PLI:	Phase Lag Index	Functional connectivity	Synchronization of activity (the consistency with which one signal is phase leading or lagging with respect to another signal), indicating the strength by which areas are functionally connected; mean of all channels	High PLI → high whole-brain FC
rC:	relative average Clustering coefficient	Local FC	Clustering coefficient: the extent to which neighbors of a node are connected to each other; Relative average clustering coefficient: the number of connections between the neighbors of a node divided by the total number of possible connections between them, averaged for all nodes, and for normalization, divided by the mean ‘average clustering coefficient’ of 50 surrogate random networks of identical density	High rC → high local FC
rL:	relative average path Length	Global FC	Path length: the number of connections between two nodes; Relative average path length: the number of connections in the shortest path to get from one node to another, averaged for all nodes, and for normalization, divided by the mean ‘average path length’ of 50 surrogate random networks of identical density	Low rL → high global FC
SWI:	Small-World Index	Entire network configuration	The extent to which rC and rL are in optimal balance (rC divided by rL)	Trade-off between a low SWI more in favor of local clustering and a high SWI more in favor of global integration
Degr:	Maximum Degree fraction	Connectivity of individual nodes	Degree fraction of a node: the number of connections that node has divided by the total number of connections; Maximum degree fraction: the highest degree fraction of all nodes	High Degr → presence of one/more hubs in the network
Ecc:	Average Eccentricity	Connectivity of individual nodes	Eccentricity of a node: the number of connections in the longest path^a^ from that node to any other node, divided by the total number of connections; Average eccentricity: mean eccentricity of all nodes in the network	Low Ecc → presence of one/more hubs in the network
BC:	Maximum Betweenness Centrality	Connectivity of individual nodes	Betweenness centrality of a node: the number of paths going through that node divided by the total number of paths^a^; Maximum betweenness centrality: the highest BC of all nodes	High BC → presence of one/more hubs in the network
Leaf:	Leaf fraction	Local FC	Leaf number: the number of nodes in the network that have only one connection (degree = 1); Leaf fraction: leaf number divided by the total number of nodes	High Leaf → high local FC
Diam:	Diameter	Global FC	The number of connections in the longest path^a^ between any two nodes in the network, and for normalization, divided by the maximum number of connections	Low Diam → high global FC
TH:	Tree Hierarchy	Entire network configuration	The balance between diameter reduction (a star-like configuration) and prevention from overloading central nodes (a line-like configuration); TH = leaf number/(2*(no. of nodes-1)*max BC)	Low TH^b^ → line-like configuration (no risk of overloading central nodes) High TH^b^ → star-like configuration (efficient FC)

*When measures were averaged for all channels or nodes, this concerned the 16 remaining channels (electrode positions) as shown in Figure 1. NA, not applicable.*

*^a^A path between any two nodes in a MST network is by definition the shortest path between these two nodes because the MST network only has one path between every two nodes (no cycles). Therefore, terms such as the ‘longest shortest path’ were avoided.*

*^b^TH close to 0 was considered to be low and TH close to 0.5 was considered to be high because TH ≥ 0.5 was not applicable in our data. See Boersma et al. (2013) for the full TH interpretation.*

### Weighted Networks

Weighted networks take the exact PLI values, the synchronization strength between signals, into account. Hence, it provides insight into all connections, weak and strong, on a continuous scale. For the visualization of weighted networks, several correlation thresholds were applied. PLI values above such threshold were included as connecting lines in a schematic representation of the brain, and PLI values below the threshold were not included in the network figure. Weighted network figures were created at the group level and at the individual level.

Network measures that quantify weighted networks are influenced by network density, that is, the number of connections, while the number of nodes is equal between participants (Van Diessen et al., 2015). Therefore, weighted network measures were normalized by dividing their absolute values by the mean values of 50 surrogate random networks of the same density. This increased the comparability between networks.

### Minimum Spanning Tree Networks

The MST (Stam et al., 2014) is a subnetwork of the weighted network. It has the same number of nodes as the weighted network (16 in our analysis), but the number of connections is limited to the number of nodes minus one (15 in our analysis). Every node gets connected to at least one other node in the MST, without forming cycles. The selection of connections to be included in the MST follows the principle of minimum cost, which is the inverse of FC strength (1/PLI value). To illustrate, the highest PLI value between any of the 16 nodes, which indicates the strongest functional connection, has the lowest cost, and is, therefore, the first to be included in the MST. Next, the connection with the second highest PLI value is included, and so forth, while taking into account the above-mentioned criteria (all nodes have to be connected, no cycles, restricted number of connections). Creation of the MST was performed by Kruskal’s algorithm (Kruskal, 1956) in BrainWave software.

As the MST is a binary network, it is independent of an (arbitrarily chosen) correlation threshold. Where a weighted network looks different for every other correlation threshold, the derived MST network always has one unique representation. Furthermore, MST networks are insensitive to the effect of network density because the number of nodes and connections remain the same. Both properties make MST networks well suited for comparisons between participants. A disadvantage of the MST network is that it includes a small portion of connections and does not provide information on weaker connections or local FC with cycles.

### Statistical Analysis

Statistical tests were performed for each research question using IBM SPSS Statistics software (version 23, release 23.0.0.0). (1) Network characteristics of patient groups were compared to those of the control groups by Mann–Whitney *U* tests. (2) Kendall’s tau-b correlation analyses were performed between preoperative network characteristics and preoperative language domain *z*-scores. (3) Network characteristics of patients with language impairment (*z*-score ≤ –1.5 on one or more language domains) were compared to those of patients without language impairment by means of Mann–Whitney *U* tests. Additionally, Degr and BC values were used to calculate which nodes were most often highly connected in patients with language impairment. (4) Kendall’s tau-b correlation analyses were performed between preoperative network characteristics and 1 year postoperative language domain *z*-scores.

## Results

### Participants

Demographic and clinical characteristics of the 15 glioma patients (matched to 15 healthy individuals) and the 10 meningioma patients (matched to nine healthy individuals) can be found in Table 3. At T2, there was a drop-out of two glioma patients, due to severe side effects of adjuvant treatment, and two meningioma patients, because they were no longer motivated to participate in the study.

**TABLE 3:** Demographic and clinical characteristics of the participants: number of participants (and percentage) or mean (and range).

	Glioma patients (*N* = 15)	Control group matched to glioma patients (*N* = 15)	Meningioma patients (*N* = 10)	Control group matched to meningioma patients (*N* = 9)
Gender – female	5 (33%)	6 (40%)	6 (60%)	4 (44%)
Mean age in years (range)	42.0 (22–60)	42.3 (20–59)	58.6 (50–69)	53.8 (46–59)
Mean education level^a^ (range)	5.3 (4–7)	5.4 (4–7)	5.4 (3–7)	5.2 (4–7)
Handedness^b^				
Right	10 (67%)	12 (80%)	9 (90%)	8 (89%)
Left	4 (27%)	3 (20%)	0	1 (11%)
Ambidextrous	1 (7%)	0	1 (10%)	0
Mean ‘diagnosis → surgery’ time in months (range)	20.8 (1.1–167.3)	NA	8.0 (2.1–49.7)	NA
Tumor histology and grade^c^				
Diffuse astrocytoma, grade II	5 (33%)	NA	NA	NA
Oligodendroglioma, grade II	10 (67%)	NA	NA	NA
Meningioma, grade I	NA	NA	10 (100%)	NA
Tumor localization, hemisphere				
Left	13 (87%)	NA	10 (100%)	NA
Right	2 (13%)	NA	0	NA
Tumor localization, lobes				
Frontal	5 (33%)	NA	6 (60%)	NA
Fronto-temporal	1 (7%)	NA	0	NA
Fronto-parietal	1 (7%)	NA	0	NA
Temporal	1 (7%)	NA	0	NA
Temporo-insular	3 (20%)	NA	0	NA
Parietal	3 (20%)	NA	3 (30%)	NA
Parieto-temporal	1 (7%)	NA	0	NA
Parieto-occipital	0	NA	1 (10%)	NA
Extent of resection^d^				
Partial: 20–89%	7 (47%)	NA	1 (10%)	NA
Subtotal: 90–99%	6 (40%)	NA	1 (10%)	NA
Total: 100%	2 (13%)	NA	8 (80%)	NA
Use of anti-epileptic drugs at T1	13 (87%)	NA	6 (60%)	NA
Use of anti-epileptic drugs at T2	12/13 (92%)	NA	7/8 (88%)	NA
Postoperative glioma treatment (chemo/radiotherapy ongoing or completed at T2)	10/13 (77%)	NA	NA	NA

*NA, not applicable; T1, before surgery; T2, one year after surgery. Some variables add up to 101% due to rounding.*

*^a^Education level was classified according to seven categories (1 = incomplete primary education; 2 = complete primary education; 3 = primary education and <2 years of low-level secondary education; 4 = complete low-level secondary education; 5 = complete average-level secondary education; 6 = complete high-level secondary education; 7 = complete university education; Verhage, 1964).*

*^b^Handedness was assessed by the Edinburgh Handedness Inventory (Oldfield, 1971).*

*^c^Based on the 2016 WHO (World Health Organisation) classification (Louis et al., 2016).*

*^d^Extent of resection was determined quantitatively by a neuroradiologist, based on pre- and postoperative tumor segmentation volumes.*

### Language Performance

#### T1 and T2

Pre- and postoperative language domain *z*-scores of the glioma patients are presented in Table 4 and the performance of the meningioma patients on language domain *z*-scores are presented in Table 5.

**TABLE 4:** Language domain *z*-scores of glioma patients at T1 and T2, including comparisons to normative data from a healthy population.

Language domain	T1: Language *z*-scores				Comparisons to the healthy population		T2: Language *z*-scores				Comparisons to the healthy population	
				
	*N*	*Mdn*	*Min*	*Max*	*Z*	*p*	*N*	*Mdn*	*Min*	*Max*	*Z*	*p*
P-Word Retrieval	15	–1.64	–10.71	0.83	–1.99	**0.023**	13	0.02	–7.18	0.88	–1.23	0.110
P-Phonology	15	–0.20	–5.28	0.95	–2.27	**0.012**	13	–0.84	–5.62	0.85	–2.20	**0.014**
P-Semantics	15	–0.60	–5.14	1.11	–1.87	**0.031**	13	–0.70	–3.99	0.91	–1.85	**0.032**
P-Grammar	15	–0.25	–8.33	0.74	–1.53	0.063	13	–0.59	–5.38	0.86	–2.41	**0.008**
C-Auditory Input	15	–0.69	–4.98	0.83	–1.77	**0.039**	13	–0.18	–3.97	0.83	–0.88	0.191
C-Visual Input	13	0.19	–1.05	0.56	0.32	0.376	12	0.38	–0.72	0.57	0.94	0.173
Reading	14	0.28	–5.28	0.28	2.67	**0.004**	13	0.28	–2.50	0.28	–0.69	0.247
Writing	13	–0.79	–6.12	0.55	–2.16	**0.016**	13	–0.79	–7.45	0.55	–2.78	**0.003**

*P, production; C, comprehension; N, sample size; Mdn, median; Min, minimum value; Max, maximum value; Z, standardized test statistic of the one-sample Wilcoxon signed rank tests; p, p-value (one-sided). Significant effects (p < 0.05) are presented in bold font.*

**TABLE 5:** Language domain *z*-scores of meningioma patients at T1 and T2, including comparisons to normative data from a healthy population.

Language domain	T1: Language *z*-scores				Comparisons to the healthy population		T2: Language *z*-scores				Comparisons to the healthy population	
				
	*N*	*Mdn*	*Min*	*Max*	*Z*	*p*	*N*	*Mdn*	*Min*	*Max*	*Z*	*p*
P-Word Retrieval	10	–0.58	–1.64	0.42	–1.89	**0.030**	8	–0.58	–8.44	0.88	–1.40	0.081
P-Phonology	10	–0.12	–1.34	1.16	–0.97	0.167	8	–0.11	–1.61	1.31	–0.14	0.445
P-Semantics	10	–0.48	–1.58	1.03	–1.27	0.102	8	0.38	–1.34	1.43	1.26	0.104
P-Grammar	10	–0.51	–1.29	–0.35	–2.81	**0.003**	8	–0.16	–3.77	0.43	–1.12	0.132
C-Auditory Input	10	0.07	–0.69	0.83	0.05	0.480	8	0.07	–1.19	0.83	0.28	0.390
C-Visual Input	7	–0.07	–1.18	0.74	0.17	0.433	6	0.30	–0.53	0.51	0.73	0.232
Reading	9	0.28	–2.50	0.28	1.73	**0.042**	8	0.28	–2.50	0.28	1.51	0.066
Writing	9	–0.79	–6.12	0.55	–1.98	**0.024**	8	–1.45	–6.12	0.55	–1.69	**0.046**

*P, production; C, comprehension; N, sample size; Mdn, median; Min, minimum value; Max, maximum value; Z, standardized test statistic of the one-sample Wilcoxon signed rank tests; p, p-value (one-sided). Significant effects (p < 0.05) are presented in bold font.*

### Functional Connectivity Networks and Language Functioning

Taking into account the various aims posed, we will address each of them separately.

### Functional Connectivity Network Characterization of Low-Grade Glioma and Meningioma Patients, Compared to the Network Organization in Healthy Individuals

#### Glioma Patients

##### T1

In the theta and alpha frequency band, there were no significant differences in the network characteristics between 15 patients and 15 healthy participants (Appendix 1).

##### T2

One year after surgery there was a significant difference in the small world index between the 13 glioma patients and the 15 healthy participants in the theta frequency band. Median of 1.102 in the patient population and 1.114 in the healthy participants (Appendix 1). For the healthy participants we have only one EEG registration which was used for both the analysis at T1 and T2 of the glioma patient group.

#### Meningioma Patients

In the preoperative setting, there were no significant differences in the network organization between the 10 meningioma patients and 9 healthy participants (Appendix 2).

### Correlation of Network Organization to Preoperative Language Functioning

#### Glioma Patients

##### Correlation Analyses

In the theta band, we observed correlations between network metrics and language production. A positive correlation between MST-Ecc and P-Word Retrieval, P-Semantics and P-Grammar was found (*T* = 0.43, *p* = 0.031; *T* = 0.40, *p* = 0.037; and *T* = 0.46, *p* = 0.019). Another positive correlation was found with the same language tests for MST-Diam (*T* = 0.45, *p* = 0.026; *T* = 0.50, *p* = 0.011; and *T* = 0.55, *p* = 0.005). A negative correlation was found between P-Word Retrieval and MST-Leaf and MST-TH (*T* = –0.42, *p* = 0.038 and *T* = –0.50, *p* = 0.012). P-Grammar correlated negatively with the PLI (*T* = –0.39, *p* = 0.047).

In language comprehension, correlations were also found. C-Visual Input correlated negatively with SWI, MST-Degr and MST-BC (*T* = –0.44, *p* = 0.04; *T* = –0.73, *p* = 0.001; and *T* = –0.44, *p* = 0.04). C-Auditory Input was negatively correlated with relative path length (*T* = –0.42, *p* = 0.035). P-Writing was negatively correlated with the PLI (*T* = –0.56, *p* = 0.016) and positively correlated with MST-Diam (*T* = 0.473, *p* = 0.042).

In the alpha band correlations were mainly found with language comprehension. C-Visual Input showed a positive correlation with MST-Ecc and MST-Diam (*T* = 0.44, *p* = 0.04 and *T* = 0.44, *p* = 0.046) and a negative correlation was found with SWI, relative clustering and MST-TH (*T* = –0.60, *p* = 0.005; *T* = –0.55, *p* = 0.022; and *T* = –0.46, *p* = 0.034). In production, only a positive correlation was found with P-Grammar (*T* = 0.39, *p* = 0.047). See Appendix 3.

#### Meningioma Patients

##### Correlation Analyses

For meningioma patients, a negative correlation in the theta band was also found for C-Visual Input with the SWI (*T* = –0.68, *p* = 0.033) (Appendix 4).

### Network Organization in the Language Impaired Patient Group

#### Glioma Patients

##### Patients With and Without Language Impairment

There were nine out of the 15 glioma patients with language impairment (*z*-score ≤ –1.5 on one or more language domains) and six out of 15 glioma patients without language impairment preoperatively. In the theta band, a lower eccentricity value (0.369 vs. 0.396, *p* = 0.036) and a less extended diameter (0.452 vs. 0.494, *p* = 0.012) was found in glioma patients with language impairments and a more star like hierarchy (0.422 vs. 0.388, *p* = 0.036) was related to patients with language impairment. No significant correlations were found in the alpha frequency band. Appendix 5 presents all comparisons

##### Connectivity of Individual Nodes in Patients With Language Impairment

Nodes (electrode positions) with the highest Degr and the highest BC indicate the most connected and most centrally positioned nodes in the MST network. In order to further investigate network characteristics of patients with language impairment, the nodes with the highest Degr and the highest BC rankings were selected for every patient with impaired language performance. Consecutively for each node, the percentage of patients with that node having the highest Degr/BC was calculated (as in Boersma et al., 2013). These percentages are given in Figure 2. (Two right-hemispheric glioma patients were not included here because they were unimpaired).

In the theta band, T5, P3, and Pz most often had the highest Degree/BC rankings, representing the most connected nodes in the MST network of glioma patients with language impairment (Figure 2A).

In the alpha band, O2, P4, and C4 most often had the highest Degree/BC rankings, representing the most connected nodes in the MST network of glioma patients with language impairment (Figure 2B).

It must be emphasized that the language impaired patient group used in this analysis is rather small.

#### Meningioma Patients

##### Patients With and Without Language Impairment

There were four out of 10 meningioma patients with language impairment and six out of 10 meningioma patients without language impairment preoperatively. In the theta band a shorter average path length was associated with patients with language impairment (0.896 vs. 0.915, *p* = 0.038).

##### Connectivity of Individual Nodes in Patients With Language Impairment

**FIGURE 3:**
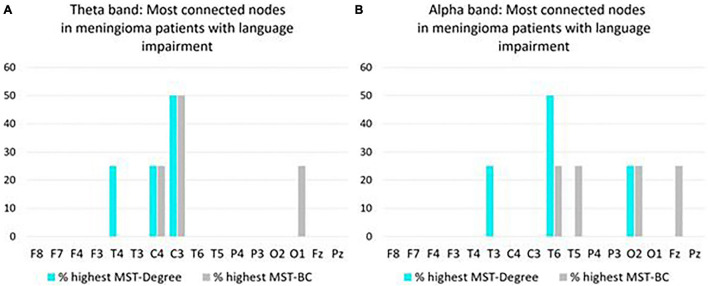
Overview of most connected nodes in MST networks in the theta band **(A)** and in the alpha band **(B)** in pre operative meningioma patients with language impairment (*N* = 4). For each node, the *y*-axis expresses the percentage of patients who have the highest Degree/Betweenness Centrality (BC) ranking for that node in their MST network. If a patient’s network has two or more nodes with a shared highest ranking, all shared highest rankings are included in the figure.

In the theta band, C3 most often had the highest Degree/BC rankings, representing the most connected node in the MST network of meningioma patients with language impairment (Figure 3A). In the alpha band, T6 most often had the highest Degree/BC rankings, representing the most connected node in the MST network of meningioma patients with language impairment (Figure 3B).

### Correlation of Preoperative Functional Connectivity Network Characteristics and Postoperative Language Outcome

#### Glioma Patients

##### Correlation Analyses

In the theta band, PLI, average clustering coefficient, MST-Ecc, MST-Degr, MST-leaf, MST-Diam, and MST-TH at T1 all are correlated with P-Word Retrieval 1 year after surgery. A positive correlation was found with MST-ECC and MST-diam (*T* = 0.69, *p* = 0.001 and *T* = 0.73, *p* = 0.001) and a negative correlation was observed with the PLI, average clustering coefficient, MST-Degr, MST-leaf and MST-TH (*T* = –0.57, *p* = 0.008; *T* = –0.44, *p* = 0.042; *T* = –0.49, *p* = 0.022; *T* = –0.57, *p* = 0.009; *T* = –0.49, *p* = 0.023). A significant correlation was found between PLI and P-Writing (*T* = –0.47, *p* = 0.036). These results indicate that poor performance on language 1 year after surgery is related to a more pronounced focal clustering and global integration on EEG in the preoperative setting.

In the alpha band, no significant correlations were found (Appendix 7).

#### Meningioma Patients

##### Correlation Analyses

In the theta band, PLI was negatively correlated with P-Word Retrieval (*T* = –0.57, *p* = 0.048) and P-Semantics (*T* = –0.57, *p* = 0.048) In the alpha band, a positive correlation was found between P-Word Retrieval and MST-Ecc (*T* = 0.57, *p* = 0.048) and MST-Diam (*T* = 0.59, *p* = 0.044). A negative correlation was observed with P-Word Retrieval and MST-Degr (*T* = –0.64, *p* = 0.026) and between P-Writing and Betweenness (*T* = –0.57, *p* = 0.014). In the alpha band, no significant correlations were found. See Appendix 8.

## Discussion

In this first study on FC networks in relation to pre- and postoperative language functioning in glioma and meningioma patients, the results will be discussed below.

### Functional Connectivity Network Characterization of Low-Grade Glioma and Meningioma Patients, Compared to the Network Organization in Healthy Individuals

#### Glioma and Meningioma Patients

The functional network in the preoperative setting of patients with the network metrics of healthy participants showed no difference in either the glioma or the meningioma patient population. It is possible that a difference could not be found by analyzing the patient group as a whole, including both patients with and without language impairment. It is possible that an evaluation in only language impaired patients could show a different network organization compared to healthy participants due to the neural plasticity which fails and give a language impairment. It is also possible that in patients without a language impairment will show a network organization which is more comparable to healthy participants because the tumor has not involved the language network or on the other hand will show differences because of the neural plasticity in which a patient will keep adequate language abilities.

Postoperative EEG was registered in glioma patients only, and a lower small-worldness was observed in the glioma patient population compared to healthy participants in the theta band. Studies on the theta band functional connectivity report increased theta-band FC before and after brain tumor surgery (Bosma et al., 2008, 2009; Van Dellen et al., 2012), particularly in the affected hemisphere (Bartolomei et al., 2006a). Higher FC in the theta band is likely to be indicative of more severe brain dysfunction, similar to brain activity in lower (delta and theta) frequency bands. This so-called ‘slow-wave activity’ during wakeful, resting conditions in adults generally represent pathological brain rhythms (Lüders and Noachtar, 2000) and it has been shown to be overrepresented in brain tumor patients (different tumor types and grades; Baayen et al., 2003; De Jongh et al., 2003; Oshino et al., 2007). When brain activity is highly present in a particular frequency band, one is more likely to find statistical interdependencies between brain signals, which provide more opportunities for high FC (Aertsen et al., 1989). Furthermore, increased theta-band FC may be related to epileptogenesis, a process by which the brain develops epilepsy. Our sample sizes were too small to verify this, but Douw et al. (2010) show that higher theta-band FC, especially in the temporal lobe, is associated with higher seizure frequency in glioma patients. The observation of the lower small-worldness in the glioma patients 1 year after surgery compared to the healthy participants may be explained by a shift toward a less optimal network organization with a higher local clustering in favor of global integration.

For the alpha band no relations were found although previous studies showed different results, Brain activity in the alpha band is representative of normal physiological brain rhythms (Lüders and Noachtar, 2000). Decreased alpha-band FC has previously been found in low-grade glioma patients (Bosma et al., 2008), and in patients with other types of brain injury, such as traumatic brain injury (Castellanos et al., 2010) and stroke (Dubovik et al., 2013). One study, however, has reported an increase of alpha-band FC in brain tumor patients (a mixed group of preoperative meningioma, low-grade glioma, and high-grade glioma patients; Bartolomei et al., 2006a). FC in higher frequency bands (beta and gamma) is generally reduced in brain tumor patients (Bartolomei et al., 2006a,b; Bosma et al., 2009; Hu et al., 2013). Hence, when the brain is affected by a tumor, FC in the alpha band may change in parallel with FC in higher frequency bands.

### Correlation of Network Organization to Preoperative Language Functioning

#### Glioma Patients

Network metrics in the glioma patient population is related to the language function, both language comprehension and production and even writing. The relations are more pronounced in the theta frequency band. In the alpha band, the relations were mainly observed with comprehension. In the theta band, hub presence and higher focal and global FC are associated with poorer language functioning before surgery. The direction of this relationship is to be expected, considering that higher theta-band FC indicates more severe brain dysfunction (previous section). Similar findings are reported by Van Dellen et al. (2012), who demonstrated that FC alterations in the theta band, such as increased local FC, were related to poorer language performance in preoperative low-grade glioma patients. This applied to category fluency performance (production-semantics at the word level), whereas the current study shows associations with grammar, production of language, and comprehension of visual input, both of which concern language functioning at the sentence level and to a lesser extent to writing.

In the alpha band, a greater small-worldness coincides with higher local clustering can be seen as a representation moving a bit to a more organized network because path length did not change that much. It is associated with poorer language comprehension before surgery. This result is inconsistent with previous findings, which demonstrated that lower global FC in the alpha band was related to poorer language production, as assessed with category fluency, in a group of preoperative low-grade and high-grade glioma patients (Derks et al., 2019). In addition, low alpha-band FC was related to poorer performance in several cognitive functions (e.g., verbal memory, information processing, and attention; Derks et al., 2019). The contrast with our result can be explained by the language task that was used to assess comprehension of visual input: sentence judgment (accuracy scores). This test was presumably too easy because it has a 50% chance of selecting the correct answer. Moreover, comprehension of visual input was the only language domain for which all patients were unimpaired preoperatively. Consequently, the correlation is based on small variation in unimpaired language performance and, therefore, may not be very informative.

#### Meningioma Patients

In the comprehension task of the meningioma patients, small-worldness again was negatively associated in the theta band. Less significant relations are presumably due to the nature of the tumor; meningiomas generally do not infiltrate the surrounding brain tissue (Wei et al., 2007). Therefore, they do not have a large direct destructive effect on brain tissue, which may be reflected by little or no FC alterations. Meningiomas can, however, cause language impairments, despite the absence of infiltrative injury (Gondar et al., 2021).

### Network Organization in the Language Impaired Patient Group

#### Glioma and Meningioma Patients

The results indicate that in glioma patients, those with language impairments in the preoperative setting show hub presence, a more global integration and more star-like configuration compared to patients without a language impairment in the theta band. In the meningioma, patients with a language impairment show a shorter median relative path length.

A more descriptive analysis of individual nodes in the MST network suggests that, in left-hemispheric tumor patients with language impairment, theta-band FC is frequently highest in left-sided brain areas and alpha-band FC is frequently highest in right-sided brain areas. This must be interpreted with caution because the analysis is based on language-impaired subgroups, and the original numbers of glioma and meningioma patients were already small.

Overall, we showed indications that FC brain networks in the preoperative setting can signify presence of language impairment in brain tumor patients. The differences show a denser pattern of functional interactions with hubs which can lead to a more optimal global organization. Maybe this pattern in the preoperative setting is already a compensatory mechanism of the brain due to the slow growth of a glioma along the white matter tracts but is still failing. In patients with non-tumor-related, but neurodegenerative language impairment (i.e., primary progressive aphasia), the localisation of altered FC in resting-state MEG has been shown to be indicative of the type of language impairment, at least distinguishing phonological, semantic, and morphosyntactic impairments (Ranasinghe et al., 2017). It is possible that investigating FC of specific brain areas can provide more accurate insights into language impairments in brain tumor patients as well. The number of patients included in this analysis is rather small and the results therefore have to interpreted with caution.

### Correlation of Preoperative Functional Connectivity Network Characteristics and Postoperative Language Outcome

#### Glioma Patients

In addition to the relation of the network metrics in the preoperative setting and language preoperatively, we also observed the same arrangement of language function 1 year after surgery, although no correction was performed on possible differences in tumor treatment or medication use. The results indicate again that poor performance on language 1 year after surgery is related to a more pronounced focal clustering and global integration on EEG in the preoperative setting. This was most pronounced with word retrieval, underlining the importance of an object naming task in the peri-operative phase of glioma surgery.

The correlation involving writing performance showed higher overall FC strength in the theta band before surgery may be associated with poorer writing after surgery. It is important to investigate this further because awake glioma surgery can induce writing impairments (Van Ierschot et al., 2018). In the current patient group, the number of preoperative versus 1 year postoperative writing impairments had doubled (Wolthuis et al., under review). Hence, the intraoperative procedure may benefit from a well-suited risk estimation on the basis of FC networks prior to surgery.

Alpha-band FC has no relation to language outcome in our study. This is in line with Lizarazu et al. (2020), who found no association between FC change in the alpha band and language change scores (object naming and semi-spontaneous speech ratings) between pre- and postoperative assessments in left-hemispheric, low-grade glioma patients. A reason for this may be that language performance did not change after surgery. The current study used language outcome scores 1 year after surgery (instead of language change scores) because performance at 1 year is most relevant for the postoperative approach, such as initiation of language intervention. To illustrate, a patient who shows no language change between pre- and postoperative assessments can either have continued unimpaired performance or persistent language impairments that require attention.

#### Meningioma Patients

Preoperative higher overall FC strength in the theta band, as well as hub presence and higher global FC in the alpha band, are associated with poorer language functioning (word retrieval, semantics, and writing) 1 year after surgery.

The functional reorganization in brain tumor patients with an impaired language performance tends to show a more pronounced local and global integration. It is possible that this configuration has arisen long before the surgery and can be seen as neural plasticity in slow growing tumors. However, this compensatory mechanism is not optimal as shown by worse language performance. It can be expected that the relation of preoperative network organization with language performance is similar to 1 year postoperatively taken into account that surgery is performed under the optimal conditions trying to preserve critical language areas.

### Limitations and Future Directions

This study is underpowered, due to the combination of small groups, many variables and, as a consequence, many statistical comparisons that accompanied our exploratory design. However, the outcomes can assist selection of variables in future studies. Language domains that appear most promising in relation to FC networks are word retrieval (as assessed with object naming), grammar, and comprehension of visual input when using a sufficiently sensitive test (e.g., combination of accuracy and speed, Mooijman et al., 2021) and to a lesser extent semantics and writing. FC network characteristics that appear most promising in relation to language functioning are MST degree, eccentricity and diameter in the theta band, and weighted small-world index in the alpha band. Although the large number of network measures complement each other in quantifying FC network configurations, a disadvantage is that they are not all independent of each other (Van Diessen et al., 2015). This is another reason for limiting the number of network measures in future research.

Functional connectivity networks in the current study may have been influenced by tumor and treatment-related factors, such as tumor location, volume, histology, use of anti-epileptic drugs, and adjuvant treatment (only applicable to glioma patients at T2), but the sample sizes were too small to control for these variables. Apart from that, there were some EEG-related challenges. EEG registration is sensitive to conductivity errors, as conductivity of the skull and other tissues can blur the signal. Consequently, signals that are registered at the scalp may not originate from the brain areas directly underneath them. Although EEG has a high temporal resolution, when compared to network studies using techniques such as fMRI, further improvement to the spatial resolution could be made by using approaches such as source modeling. Moreover, electrical conduction properties of the human head vary between individuals. This results in differences in total FC strength, as measured with the PLI. We normalized the other weighted FC network measures to increase comparability between networks, although network comparisons are still not completely unbiased by using this method (Stam et al., 2014). MEG may be preferred to EEG for FC network investigations, because it will reduce the previous problems and increase spatial resolution. However, it is more expensive and less suitable for clinical use than EEG. Overall, future studies on this topic are likely to benefit from larger sample sizes and fewer variables, carefully selected on the basis of previous work.

## Conclusion

Functional connectivity and network metrics in glioma patients and to a lesser extent in meningioma patients show significant correlations which all have the same direction. The most remarkable correlations were observed in the theta band. Hub presence and higher local and global FC are associated with poorer language functioning before surgery in glioma patients. In this exploratory study, these parameters predict worse language performance 1 year after surgery, although we have to be cautious about this finding due to a number of known and unknown patient and tumor related factors which could have influenced the results.

In meningioma patients, a greater small-worldness was related to language performance and hub presence, better average clustering and global integration were predictive of worse outcome on language function 1 year after surgery. The average eccentricity, diameter and tree hierarchy seems to be the network metrics with the more pronounced relation to language performance.

Overall, we present the first indications that FC networks are informative for language functioning in glioma patients undergoing surgery, but not (yet) to an extent that the outcomes are clinically applicable. Continuation of this line of research, especially the search for predictors of language outcome, has the potential to improve perisurgical patient care, such as treatment planning, counseling, and language rehabilitation.

## Data Availability Statement

The original contributions presented in the study are included in the article/Supplementary Material, further inquiries can be directed to the corresponding author/s.

## Ethics Statement

The studies involving human participants were reviewed and approved by Medical Ethical Review Board University Medical Centre Groningen and Medical Ethical Review Board of Erasmus Medical Center in Rotterdam. The patients/participants provided their written informed consent to participate in this study.

## Author Contributions

RB, NW, DS, PC, IB, and WV contributed to conception and design of the study. NW organized the database, performed the statistical analysis, and wrote the first draft of the manuscript. RB, NW, DS, PC, and IB wrote sections of the manuscript. All authors contributed to manuscript revision, read, and approved the submitted version.

## Conflict of Interest

The authors declare that the research was conducted in the absence of any commercial or financial relationships that could be construed as a potential conflict of interest.

## Publisher’s Note

All claims expressed in this article are solely those of the authors and do not necessarily represent those of their affiliated organizations, or those of the publisher, the editors and the reviewers. Any product that may be evaluated in this article, or claim that may be made by its manufacturer, is not guaranteed or endorsed by the publisher.

## Footnotes

1 The interpretation of FC network configurations is dependent on the investigated frequency band; brain activity in the delta and theta bands is considered pathological (this is usually absent in healthy adults while awake), whereas brain activity in the alpha and beta bands indicates normal physiological brain rhythms (Niedermeyer, 1999).

2 The interpretation of FC network configurations is dependent on the investigated frequency band; brain activity in the delta and theta bands is considered pathological (this is usually absent in healthy adults while awake), whereas brain activity in the alpha and beta bands indicates normal physiological brain rhythms (Niedermeyer, 1999).

3 Verbal fluency, in the form of category or letter fluency, is classified under language functioning instead of executive functioning here because the tests at least partly assess language processing (Whiteside et al., 2016; Aita et al., 2019).

4 Edinburgh handedness inventory was used for handedness.

## References

[B1] Aertsen A. M. H. J. Gerstein G. L. Habib M. K. Palm G. (1989). Dynamics of neuronal firingcorrelation: modulation of “effective connectivity.” *J. Neurophysiol.* 61 900–917. 10.1152/jn.1989.61.5.900 2723733

[B2] Aita S. L. Beach J. D. Taylor S. E. Borgogna N. C. Harrell M. N. Hill B. D. (2019). Executive, language, or both? An examination of the construct validity of verbal fluency measures. *Appl. Neuropsychol.* 26 441–451. 10.1080/23279095.2018.1439830 29513079

[B3] Antonsson M. Jakola A. Longoni F. Carstam L. Hartelius L. Thordstein M. (2018). Post-surgical effects on language in patients with presumed low_grade glioma. *Acta Neurol. Scand.* 137 469–480. 10.1111/ane.12887 29265169

[B4] Baayen J. C. De Jongh A. Stam C. J. De Munck J. C. Jonkman J. J. Kasteleijn-Nolst Trenité D. G. A. (2003). Localization of slow wave activity in patients with tumor-associated epilepsy. *Brain Topogr.* 16 85–93. 10.1023/B:BRAT.0000006332.71345.b7 14977201

[B5] Bartolomei F. Bosma I. Klein M. Baayen J. C. Reijneveld J. C. Postma T. J. (2006a). Disturbed functional connectivity in brain tumour patients: evaluation by graph analysis of synchronization matrices. *Clin. Neurophysiol.* 117 2039–2049. 10.1016/j.clinph.2006.05.018 16859985

[B6] Bartolomei F. Bosma I. Klein M. Baayen J. C. Reijneveld J. C. Postma T. J. (2006b). How do brain tumors alter functional connectivity? A magnetoencephalography study. *Ann. Neurol.* 59 128–138. 10.1002/ana.20710 16278872

[B7] Boersma M. Smit D. J. A. Boomsma D. I. De Geus E. J. C. Delemarre-van de Waal H. A. Stam C. J. (2013). Growing trees in child brains: graph theoretical analysis of electroencephalography-derived minimum spanning tree in 5- and 7-year-old children reflects brain maturation. *Brain Connect.* 3 50–60. 10.1089/brain.2012.0106 23106635

[B8] Bosma I. Douw L. Bartolomei F. Heimans J. J. Van Dijk B. W. Postma T. J. (2008). Synchronized brain activity and neurocognitive function in patients with low-grade glioma: a magnetoencephalography study. *Neuro Oncol.* 10 734–744. 10.1215/15228517-2008-034 18650489 PMC2666250

[B9] Bosma I. Reijneveld J. C. Klein M. Douw L. Van Dijk B. W. Heimans J. J. (2009). Disturbed functional brain networks and neurocognitive function in low-grade glioma patients: a graph theoretical analysis of resting-state MEG. *Nonlinear Biomed. Phys.* 3:9. 10.1186/1753-4631-3-9 19698149 PMC2745411

[B10] Brain Products GmbH (2013). *Brain Products GmbH, Solutions for Neurophysiological Research.* Available online at: brainproducts.com

[B11] Castellanos N. P. Paúl N. Ordóñez V. E. Demuynck O. Bajo R. Campo P. (2010). Reorganization of functional connectivity as a correlate of cognitive recovery in acquired brain injury. *Brain* 133 2365–2381. 10.1093/brain/awq174 20826433

[B12] Davie G. L. Hutcheson K. A. Barringer D. A. Weinberg J. S. Lewin J. S. (2009). Aphasia in patients after brain tumour resection. *Aphasiology* 23 1196–1206. 10.1080/02687030802436900

[B13] De Jongh A. Baayen J. C. De Munck J. C. Heethaar R. M. Vandertop W. P. Stam C. J. (2003). The influence of brain tumor treatment on pathological delta activity in MEG. *NeuroImage* 20 2291–2301. 10.1016/j.neuroimage.2003.07.030 14683730

[B14] De Renzi E. Faglioni P. (1978). Normative data and screening power of a shortened version of the token test. *Cortex* 14 41–49. 10.1016/S0010-9452(78)80006-9 16295108

[B15] De Witte E. Satoer D. Robert E. Colle H. Verheyen S. Visch-Brink E. (2015). The dutch linguistic intraoperative protocol: a valid linguistic approach to awake brain surgery. *Brain Lang.* 140 35–48. 10.1016/j.bandl.2014.10.011 25526520

[B16] Derks J. Kulik S. Wesseling P. Numan T. Hillebrand A. van Dellen E. (2019). Understanding cognitive functioning in glioma patients: the relevance of IDH-mutation status and functional connectivity. *Brain Behav.* 9 1–9. 10.1002/brb3.1204 30809977 PMC6456787

[B17] Deverdun J. van Dokkum L. E. H. Le Bars E. Herbet G. Mura T. Moritz S. (2020). Languagereorganization after resection of low-grade gliomas, an fMRI task based connectivity study. *Brain Imaging Behav.* 14 1779–1791. 31111301 10.1007/s11682-019-00114-7

[B18] Di Cristofori A. Zarino B. Bertani G. Locatelli M. Rampini P. Carrabba G. (2018). Surgery in elderly patients with intracranial meningioma: neuropsychological functioning during a long term follow-up. *J. Neuro Oncol.* 137 611–619. 10.1007/s11060-018-2754-3 29330748

[B19] Douw L. Van Dellen E. De Groot M. Heimans J. J. Klein M. Stam C. J. (2010). Epilepsy is related to theta band brain connectivity and network topology in brain tumor patients. *BMC Neurosci.* 11:103. 10.1186/1471-2202-11-103 20731854 PMC2936439

[B20] Dubovik S. Ptak R. Aboulafia T. Magnin C. Gillabert N. Allet L. (2013). EEG alpha band synchrony predicts cognitive and motor performance in patients with ischemic stroke. *Behav. Neurol.* 26 187–189. 10.3233/BEN-2012-129007 22713421 PMC5214220

[B21] Gondar R. Patet G. Schaller K. Meling T. R. (2021). Meningiomas and cognitive impairment after treatment: a systematic and narrative review. *Cancers* 13:1846. 10.3390/cancers13081846 33924372 PMC8070481

[B22] Hilari K. Needle J. J. Harrison K. L. (2012). What are the important factors in health-related quality of life for people with aphasia? A systematic review. *Arch. Phys. Med. Rehabilit.* 93 S86–S95. 10.1016/j.apmr.2011.05.028 22119074

[B23] Ho V. K. Y. Reijneveld J. C. Enting R. H. Bienfait H. P. Robe P. Baumert B. G. (2014). Changing incidence and improved survival of gliomas. *Eur. J. Cancer* 50 2309–2318. 10.1016/j.ejca.2014.05.019 24972545

[B24] Hu X. H. Lei T. Xu H. Z. Zou Y. J. Liu H. Y. (2013). Resting-state magnetoencephalography study of “small world” characteristics and cognitive dysfunction in patients with glioma. *Onco Targets Ther.* 6 311–313. 10.2147/OTT.S42471 23579278 PMC3621646

[B25] Jin L. Li C. Zhang Y. Yuan T. Ying J. Zuo Z. (2021). The functional reorganization of language network modules in glioma patients: new insights from resting state fMRI study. *Front. Oncol.* 11:617179. 10.3389/fonc.2021.617179 33718172 PMC7953055

[B26] Kruskal J. B. (1956). On the shortest spanning subtree of a graph and the traveling salesman problem. *Proc. Am. Math. Soc.* 7 48–50. 10.1090/S0002-9939-1956-0078686-7 30656504

[B27] Lizarazu M. Gil-Robles S. Pomposo I. Nara S. Amoruso L. Quiñones I. (2020). Spatiotemporal dynamics of postoperative functional plasticity in patients with brain tumors in language areas. *Brain Lang.* 202:104741. 10.1016/j.bandl.2019.104741 31931399

[B28] Louis D. N. Perry A. Reifenberger G. von Deimling A. Figarella-Branger D. Cavenee W. K. (2016). The 2016 world health organization classification of tumors of the central nervous system: a summary. *Acta Neuropathol.* 131 1–18. 10.1007/s00401-016-1545-1 27157931

[B29] Lüders H. O. Noachtar S. (2000). *Atlas And Classification Of Electroencephalography.* Philadelphia, PA: W. B. Saunders.

[B30] Luteijn F. Barelds D. P. F. (2004). *Groninger Intelligentie Test – 2 (GIT-2).* Amsterdam: Pearson.

[B31] Mooijman S. Bos L. S. Witte De E. Vincent A. Visch-Brink E. Satoer D. (2021). Language processing in glioma patients: speed or accuracy as a sensitive measure? *Aphasiology* 1–25. 10.1080/02687038.2021.1970099 [Epub ahead of print].

[B32] Nicolo P. Rizk S. Magnin C. Di Pietro M. Schnider A. Guggisberg A. G. (2015). Coherent neural oscillations predict future motor and language improvement after stroke. *Brain* 138 3048–3060. 10.1093/brain/awv200 26163304

[B33] Niedermeyer E. (1999). “The normal EEG of the waking adult,” in *Electroencephalography: Basic Principles, Clinical Applications, And Related Fields*, 4th Edn, eds Niedermeyer E. Lopes da Silva F. (Philadelphia, PA: Williams and Wilkins), 149–173.

[B34] Oldfield R. C. (1971). The assessment and analysis of handedness: the edinburgh inventory. *Neuropsychologia* 9 97–113. 10.1016/0028-3932(71)90067-4 5146491

[B35] Oshino S. Kato A. Wakayama A. Taniguchi M. Hirata M. Yoshimine T. (2007). Magnetoencephalographic analysis of cortical oscillatory activity in patients with brain tumors: synthetic aperture magnetometry (SAM) functional imaging of delta band activity. *NeuroImage* 34 957–964. 10.1016/j.neuroimage.2006.08.054 17175174

[B36] Ranasinghe K. G. Hinkley L. B. Beagle A. J. Mizuiri D. Honma S. M. Welch A. E. (2017). Distinct spatiotemporal patterns of neuronal functional connectivity in primary progressive aphasia variants. *Brain* 140 2737–2751. 10.1093/brain/awx217 28969381 PMC5841154

[B37] Rofes A. (2012). *The Verb In Sentence Context Test: Standardization And Application In Awake Neurosurgery.* Ph.D. Dissertation. Groningen: University of Groningen.

[B38] Santini B. Talacchi A. Squintani G. Casagrande F. Capasso R. Miceli G. (2012). Cognitive outcome after awake surgery for tumors in language areas. *J. Neuro Oncol.* 108 319–326. 10.1007/s11060-012-0817-4 22350433

[B39] Satoer D. De Witte E. Bastiaanse R. Vincent A. Mariën P. Visch-Brink E. (2019). “Diagnostic Instrument for mild aphasia (DIMA): standardization and clinical application,” in *Proceedings of the Conference Abstract: Academy of Aphasia 55th Annual Meeting*, Baltimore. 10.3389/conf.fnhum.2017.223.00103

[B40] Schmand B. Groenink S. C. Van den Dungen M. (2008). Letterfluency: psychometrische eigenschappen en nederlandse normen. *Tijdschr. Gerontol. Geriatr.* 39 65–77. 10.1007/BF03078128 18500167

[B41] Stam C. J. (2018). *BrainWave.* Available online at: http://home.kpn.nl/stam7883/brainwave.html (accessed 2020).

[B42] Stam C. J. Nolte G. Daffertshofer A. (2007). Phase lag index: assessment of functional connectivity from multi channel EEG and MEG with diminished bias from common sources. *Hum. Brain Mapping* 28 1178–1193. 10.1002/hbm.20346 17266107 PMC6871367

[B43] Stam C. J. Tewarie P. Van Dellen E. Van Straaten E. C. W. Hillebrand A. Van Mieghem P. (2014). The trees and the forest: characterization of complex brain networks with minimum spanning trees. *Int. J. Psychophysiol.* 92 129–138. 10.1016/j.ijpsycho.2014.04.001 24726900

[B44] Van Alkemade H. De Leau M. Dieleman E. M. T. Kardaun J. W. P. F. Van Os R. Vandertop W. P. (2012). Impaired survival and long-term neurological problems in benign meningioma. *Neuro Oncol.* 14 658–666. 10.1093/neuonc/nos013 22406926 PMC3337301

[B45] Van Dellen E. De Witt Hamer P. C. Douw L. Klein M. Heimans J. J. Stam C. J. (2013). Connectivity in MEG resting-state networks increases after resective surgery for low-grade glioma and correlates with improved cognitive performance. *NeuroImage Clin.* 2 1–7. 10.1016/j.nicl.2012.10.007 24179752 PMC3777771

[B46] Van Dellen E. Douw L. Hillebrand A. Ris-Hilgersom I. H. M. Schoonheim M. M. Baayen J. C. (2012). MEG network differences between low- and high-grade glioma related to epilepsy and cognition. *PLoS One* 7:e50122. 10.1371/journal.pone.0050122 23166829 PMC3498183

[B47] Van Diessen E. Numan T. Van Dellen E. Van der Kooi A. W. Boersma M. Hofman D. (2015). Opportunities and methodological challenges in EEG and MEG resting state functional brain network research. *Clin. Neurophysiol.* 126 1468–1481. 10.1016/j.clinph.2014.11.018 25511636

[B48] Van Ierschot F. (2018). *Written Language In Awake Surgery – Monitoring Of Reading And Spelling In Glioma Patients Undergoing Awake Surgery.* Available online at: https://nvneuropsy.nl/uploads/prijzen/inzendingen/5abe08aaf035957a7a9e38858a3ce0b7.pdf (accessed 2020).

[B49] Van Ierschot F. Bastiaanse R. Miceli G. (2018). Evaluating spelling in glioma patients undergoing awake surgery: a systematic review. *Neuropsychol. Rev.* 28 470–495. 10.1007/s11065-018-9391-7 30578451

[B50] Van Kessel E. Snijders T. J. Baumfalk A. E. Ruis C. van Baarsen K. M. Broekman M. L. (2020). Neurocognitive changes after awake surgery in glioma patients: a retrospective cohort study. *J. Neuro Oncol.* 146 97–109. 10.1007/s11060-019-03341-6 31802314 PMC6938472

[B51] Van Nieuwenhuizen D. Douw L. Klein M. Peerdeman S. M. Heimans J. J. Reijneveld J. C. (2018). Cognitive functioning and functional brain networks in postoperative WHO grade I meningioma patients. *J. Neuro Oncol.* 140 605–613. 10.1007/s11060-018-2987-1 30219943 PMC6267232

[B52] Van Straaten E. C. W. Stam C. J. (2013). Structure out of chaos: Functional brain network analysis with EEG, MEG, and functional MRI. *Eur. Neuropsychopharmacol.* 23 7–18. 10.1016/j.euroneuro.2012.10.010 23158686

[B53] Verhage F. (1964). *Intelligentie En Leeftijd: Onderzoek BIJ Nederlanders Van 12 TOT 77 JAAR.* Assen: Van Gorkum.

[B54] Watts D. J. Strogatz S. H. (1998). Collective dynamics of small-world networks. *Nature* 393 440–442. 9623998 10.1038/30918

[B55] Wei C. W. Guo G. Mikulis D. J. (2007). Tumor effects on cerebral white matter as characterized by diffusion tensor tractography. *Can. J. Neurol. Sci.* 34 62–68. 10.1017/s0317167100005801 17352349

[B56] Whiteside D. M. Kealey T. Semla M. Luu H. Rice L. Basso M. R. (2016). Verbal fluency: language or executive function measure? *Appl. Neuropsychol.* 23 29–34. 10.1080/23279095.2015.1004574 26111011

[B57] Zhang X. Lei X. Wu T. Jiang T. (2014). A review of EEG and MEG for brainnetome research. *Cogn. Neurodyn.* 8 87–98. 10.1007/s11571-013-9274-9 24624229 PMC3945460

