# Supplementary material for: Resting-State Electroencephalography Functional Connectivity Networks Relate to Pre- and Postoperative Language Functioning in Low-Grade Glioma and Meningioma Patients
Source: Front Neurosci. 2021 Dec 8;15:785969. doi: 10.3389/fnins.2021.785969 (PMC8693574; doi:10.3389/fnins.2021.785969)
Supplement: Supplementary file 1 [file Table_1.docx]

**Appendix 1 – FC network characteristics: glioma patients vs. healthy individuals**

**T1: before surgery**

*Theta- and alpha-band FC network characteristics in preoperative glioma patients compared to healthy individuals*

|  | Glioma patients at T1  (*N* = 15) | | |  | Healthy individuals  (*N* = 15) | | |  | Comparisons | |
| --- | --- | --- | --- | --- | --- | --- | --- | --- | --- | --- |
|  | *Mdn* | *Min* | *Max* |  | *Mdn* | *Min* | *Max* |  | *U* | *p* |
| **Theta band** |  |  |  |  |  |  |  |  |  |  |
| W-PLI | 0.123 | 0.107 | 0.207 |  | 0.130 | 0.100 | 0.153 |  | 112.0 | 1.000 |
| W-rC | 0.998 | 0.978 | 1.106 |  | 1.023 | 0.974 | 1.073 |  | 99.0 | 0.595 |
| W-rL | 0.901 | 0.884 | 0.955 |  | 0.909 | 0.887 | 0.950 |  | 89.0 | 0.345 |
| W-SWI | 1.106 | 1.089 | 1.173 |  | 1.114 | 1.090 | 1.149 |  | 103.0 | 0.713 |
| MST-Degr | 0.347 | 0.280 | 0.427 |  | 0.333 | 0.293 | 0.427 |  | 100.0 | 0.624 |
| MST-Ecc | 0.375 | 0.330 | 0.439 |  | 0.373 | 0.312 | 0.433 |  | 111.0 | 0.967 |
| MST-BC | 0.728 | 0.657 | 0.796 |  | 0.726 | 0.694 | 0.787 |  | 109.0 | 0.902 |
| MST-Leaf | 0.600 | 0.453 | 0.627 |  | 0.573 | 0.493 | 0.640 |  | 95.0 | 0.486 |
| MST-Diam | 0.467 | 0.400 | 0.547 |  | 0.467 | 0.387 | 0.533 |  | 109.0 | 0.902 |
| MST-TH | 0.398 | 0.311 | 0.439 |  | 0.398 | 0.330 | 0.452 |  | 90.0 | 0.367 |
| **Alpha band** |  |  |  |  |  |  |  |  |  |  |
| W-PLI | 0.142 | 0.103 | 0.245 |  | 0.185 | 0.104 | 0.495 |  | 74.0 | 0.116 |
| W-rC | 1.012 | 0.973 | 1.083 |  | 1.015 | 0.970 | 1.101 |  | 105.0 | 0.775 |
| W-rL | 0.907 | 0.887 | 0.957 |  | 0.917 | 0.888 | 0.936 |  | 97.0 | 0.539 |
| W-SWI | 1.119 | 1.077 | 1.188 |  | 1.119 | 1.065 | 1.181 |  | 106.0 | 0.806 |
| MST-Degr | 0.347 | 0.293 | 0.480 |  | 0.360 | 0.293 | 0.493 |  | 96.5 | 0.512 |
| MST-Ecc | 0.374 | 0.312 | 0.420 |  | 0.381 | 0.299 | 0.427 |  | 102.0 | 0.683 |
| MST-BC | 0.714 | 0.673 | 0.819 |  | 0.731 | 0.653 | 0.813 |  | 97.0 | 0.539 |
| MST-Leaf | 0.613 | 0.520 | 0.680 |  | 0.587 | 0.507 | 0.720 |  | 111.5 | 0.967 |
| MST-Diam | 0.467 | 0.387 | 0.520 |  | 0.480 | 0.373 | 0.547 |  | 104.0 | 0.744 |
| MST-TH | 0.403 | 0.378 | 0.475 |  | 0.398 | 0.352 | 0.486 |  | 104.5 | 0.744 |

*Note*. *Mdn* = median; *Min* = minimum value; *Max* = maximum value; *U* = test statistic of the Mann-Whitney U tests; *p* = p-value (two-sided). W = weighted: these network measures quantify weighted FC networks; MST = Minimum Spanning Tree: these network measures quantify Minimum Spanning Tree FC networks. FC = functional connectivity; PLI = Phase lag index, mean of all 16 remaining electrodes; rC = relative average clustering coefficient; rL = relative average path length; SWI = small-world index; MST-Degr = MST-maximum degree; MST-Ecc = MST-eccentricity, mean of all nodes; MST-BC = MST-maximum betweenness centrality; MST-Leaf = MST-leaf fraction; MST-Diam = MST-diameter; MST-TH = MST-tree hierarchy.

**T2: 1 year after surgery**

*Theta- and alpha-band FC network characteristics in 1 year postoperative glioma patients compared to healthy individuals*

|  | Glioma patients at T2  (*N* = 11) | | |  | Healthy individuals  (*N* = 15) | | |  | Comparisons | |
| --- | --- | --- | --- | --- | --- | --- | --- | --- | --- | --- |
|  | *Mdn* | *Min* | *Max* |  | *Mdn* | *Min* | *Max* |  | *U* | *p* |
| **Theta band** |  |  |  |  |  |  |  |  |  |  |
| W-PLI | 0.133 | 0.121 | 0.209 |  | 0.130 | 0.100 | 0.153 |  | 65.0 | 0.384 |
| W-rC | 1.002 | 0.979 | 1.058 |  | 1.023 | 0.974 | 1.073 |  | 63.0 | 0.330 |
| W-rL | 0.908 | 0.881 | 0.965 |  | 0.909 | 0.887 | 0.950 |  | 80.0 | 0.919 |
| W-SWI | 1.102 | 1.083 | 1.152 |  | 1.114 | 1.090 | 1.149 |  | 42.0 | *0.036* |
| MST-Degr | 0.320 | 0.267 | 0.360 |  | 0.333 | 0.293 | 0.427 |  | 58.5 | 0.217 |
| MST-Ecc | 0.397 | 0.347 | 0.446 |  | 0.373 | 0.312 | 0.433 |  | 59.0 | 0.237 |
| MST-BC | 0.707 | 0.669 | 0.773 |  | 0.726 | 0.694 | 0.787 |  | 47.5 | 0.069 |
| MST-Leaf | 0.533 | 0.493 | 0.627 |  | 0.573 | 0.493 | 0.640 |  | 56.0 | 0.180 |
| MST-Diam | 0.493 | 0.427 | 0.560 |  | 0.467 | 0.387 | 0.533 |  | 59.5 | 0.237 |
| MST-TH | 0.375 | 0.329 | 0.450 |  | 0.398 | 0.330 | 0.452 |  | 74.0 | 0.683 |
| **Alpha band** |  |  |  |  |  |  |  |  |  |  |
| W-PLI | 0.139 | 0.095 | 0.245 |  | 0.185 | 0.104 | 0.495 |  | 50.0 | 0.097 |
| W-rC | 1.029 | 0.964 | 1.080 |  | 1.015 | 0.970 | 1.101 |  | 77.0 | 0.799 |
| W-rL | 0.916 | 0.891 | 0.949 |  | 0.917 | 0.888 | 0.936 |  | 77.0 | 0.799 |
| W-SWI | 1.126 | 1.049 | 1.181 |  | 1.119 | 1.065 | 1.181 |  | 77.0 | 0.799 |
| MST-Degr | 0.347 | 0.280 | 0.413 |  | 0.360 | 0.293 | 0.493 |  | 62.0 | 0.305 |
| MST-Ecc | 0.386 | 0.333 | 0.422 |  | 0.381 | 0.299 | 0.427 |  | 69.0 | 0.507 |
| MST-BC | 0.716 | 0.646 | 0.771 |  | 0.731 | 0.653 | 0.813 |  | 73.0 | 0.646 |
| MST-Leaf | 0.587 | 0.520 | 0.653 |  | 0.587 | 0.507 | 0.720 |  | 79.0 | 0.878 |
| MST-Diam | 0.480 | 0.413 | 0.520 |  | 0.480 | 0.373 | 0.547 |  | 58.0 | 0.217 |
| MST-TH | 0.403 | 0.376 | 0.438 |  | 0.398 | 0.352 | 0.486 |  | 72.5 | 0.610 |

*Note*. *Mdn* = median; *Min* = minimum value; *Max* = maximum value; *U* = test statistic of the Mann-Whitney U tests; *p* = p-value (two-sided). Comparisons with *p* < 0.05 are presented in italics. W = weighted: these network measures quantify weighted FC networks; MST = Minimum Spanning Tree: these network measures quantify Minimum Spanning Tree FC networks. FC = functional connectivity; PLI = Phase lag index, mean of all 16 remaining electrodes; rC = relative average clustering coefficient; rL = relative average path length; SWI = small-world index; MST-Degr = MST-maximum degree; MST-Ecc = MST-eccentricity, mean of all nodes; MST-BC = MST-maximum betweenness centrality; MST-Leaf = MST-leaf fraction; MST-Diam = MST-diameter; MST-TH = MST-tree hierarchy.
