# Supplementary material for: Resting-State Electroencephalography Functional Connectivity Networks Relate to Pre- and Postoperative Language Functioning in Low-Grade Glioma and Meningioma Patients
Source: Front Neurosci. 2021 Dec 8;15:785969. doi: 10.3389/fnins.2021.785969 (PMC8693574; doi:10.3389/fnins.2021.785969)
Supplement: Supplementary file 2 [file Table_2.docx]

**Appendix 2 – FC network characteristics: meningioma patients vs. healthy individuals**

**T1: before surgery**

*Theta- and alpha-band FC network characteristics in preoperative meningioma patients compared to healthy individuals*

|  | Meningioma patients  at T1  (*N* = 10) | | |  | Healthy individuals  (*N* = 9) | | |  | Comparisons | |
| --- | --- | --- | --- | --- | --- | --- | --- | --- | --- | --- |
|  | *Mdn* | *Min* | *Max* |  | *Mdn* | *Min* | *Max* |  | *U* | *p* |
| **Theta band** |  |  |  |  |  |  |  |  |  |  |
| W-PLI | 0.134 | 0.113 | 0.449 |  | 0.129 | 0.100 | 0.153 |  | 37.0 | 0.549 |
| W-rC | 1.004 | 0.952 | 1.061 |  | 1.019 | 0.974 | 1.029 |  | 40.0 | 0.720 |
| W-rL | 0.904 | 0.888 | 1.012 |  | 0.905 | 0.893 | 0.922 |  | 44.0 | 0.968 |
| W-SWI | 1.097 | 1.051 | 1.156 |  | 1.112 | 1.091 | 1.136 |  | 30.0 | 0.243 |
| MST-Degr | 0.333 | 0.267 | 0.547 |  | 0.334 | 0.320 | 0.400 |  | 42.0 | 0.842 |
| MST-Ecc | 0.378 | 0.296 | 0.429 |  | 0.384 | 0.322 | 0.422 |  | 39.5 | 0.661 |
| MST-BC | 0.722 | 0.707 | 0.874 |  | 0.729 | 0.694 | 0.762 |  | 44.0 | 0.968 |
| MST-Leaf | 0.540 | 0.453 | 0.653 |  | 0.560 | 0.493 | 0.640 |  | 41.5 | 0.780 |
| MST-Diam | 0.474 | 0.360 | 0.547 |  | 0.480 | 0.400 | 0.533 |  | 40.5 | 0.720 |
| MST-TH | 0.375 | 0.321 | 0.435 |  | 0.402 | 0.330 | 0.427 |  | 39.0 | 0.661 |
| **Alpha band** |  |  |  |  |  |  |  |  |  |  |
| W-PLI | 0.169 | 0.107 | 0.274 |  | 0.251 | 0.122 | 0.495 |  | 27.0 | 0.156 |
| W-rC | 1.015 | 0.976 | 1.068 |  | 1.001 | 0.970 | 1.057 |  | 37.0 | 0.549 |
| W-rL | 0.909 | 0.889 | 0.944 |  | 0.917 | 0.888 | 0.927 |  | 45.0 | 1.000 |
| W-SWI | 1.121 | 1.045 | 1.160 |  | 1.102 | 1.065 | 1.160 |  | 37.0 | 0.549 |
| MST-Degr | 0.347 | 0.280 | 0.493 |  | 0.373 | 0.307 | 0.440 |  | 38.0 | 0.604 |
| MST-Ecc | 0.361 | 0.311 | 0.459 |  | 0.381 | 0.331 | 0.427 |  | 42.0 | 0.842 |
| MST-BC | 0.738 | 0.670 | 0.798 |  | 0.735 | 0.684 | 0.808 |  | 42.5 | 0.842 |
| MST-Leaf | 0.587 | 0.480 | 0.693 |  | 0.587 | 0.507 | 0.693 |  | 44.0 | 0.968 |
| MST-Diam | 0.447 | 0.373 | 0.587 |  | 0.480 | 0.400 | 0.547 |  | 45.0 | 1.000 |
| MST-TH | 0.401 | 0.350 | 0.464 |  | 0.393 | 0.361 | 0.486 |  | 44.0 | 0.968 |

*Note*. *Mdn* = median; *Min* = minimum value; *Max* = maximum value; *U* = test statistic of the Mann-Whitney U tests; *p* = p-value (two-sided). W = weighted: these network measures quantify weighted FC networks; MST = Minimum Spanning Tree: these network measures quantify Minimum Spanning Tree FC networks. FC = functional connectivity; PLI = Phase lag index, mean of all 16 remaining electrodes; rC = relative average clustering coefficient; rL = relative average path length; SWI = small-world index; MST-Degr = MST-maximum degree; MST-Ecc = MST-eccentricity, mean of all nodes; MST-BC = MST-maximum betweenness centrality; MST-Leaf = MST-leaf fraction; MST-Diam = MST-diameter; MST-TH = MST-tree hierarchy.
