# Supplementary material for: Resting-State Electroencephalography Functional Connectivity Networks Relate to Pre- and Postoperative Language Functioning in Low-Grade Glioma and Meningioma Patients
Source: Front Neurosci. 2021 Dec 8;15:785969. doi: 10.3389/fnins.2021.785969 (PMC8693574; doi:10.3389/fnins.2021.785969)
Supplement: Supplementary file 3 [file Table_3.DOCX]

**Appendix 3 – FC network characteristics at T1 and language at T1 in glioma patients**

*Correlation analyses between FC network measures at T1 and language domain z-scores at T1 in glioma patients*

|  |  | W-PLI | W-  rC | W-  rL | W-SWI | MST-Degr | MST-Ecc | MST-BC | MST-Leaf | MST-Diam | MST-TH |
| --- | --- | --- | --- | --- | --- | --- | --- | --- | --- | --- | --- |
| **Theta band** |  |  |  |  |  |  |  |  |  |  |  |
| P-Word Retrieval  (*N* = 15) | *ᴛ* | - | - | - | - | - | 0.43 | - | -0.42 | 0.45 | -0.50 |
|  | *p* |  |  |  |  |  | .031 |  | .038 | .026 | .012 |
| P-Phonology  (*N* = 15) | *ᴛ* | - | - | - | - | - | - | - | - | - | - |
|  | *p* |  |  |  |  |  |  |  |  |  |  |
| P-Semantics  (*N* = 15) | *ᴛ* | - | - | - | - | - | 0.40 | - | - | 0.50 | - |
|  | *p* |  |  |  |  |  | .037 |  |  | .011 |  |
| P-grammar  (*N* = 15) | *ᴛ* | -0.39 | - | - | - | - | 0.46 | - | - | 0.55 | - |
|  | *p* | .047 |  |  |  |  | .019 |  |  | .005 |  |
| C-Auditory Input  (*N* = 15) | *ᴛ* | - | - | -0.42 | - | - | - | - | - | - | - |
|  | *p* |  |  | .035 |  |  |  |  |  |  |  |
| C-Visual Input  (*N* = 13) | *ᴛ* | - | - | - | -0.44 | -0.73 | - | -0.44 | - | - | - |
|  | *p* |  |  |  | .040 | .001 |  | .040 |  |  |  |
| Reading  (*N* = 14) | *ᴛ* | - | - | - | - | - | - | - | - | - | - |
|  | *p* |  |  |  |  |  |  |  |  |  |  |
| Writing  (*N* = 13) | *ᴛ* | -0.56 | - | - | - | - | - | - | - | 0.47 | - |
|  | *p* | .016 |  |  |  |  |  |  |  | .042 |  |
| **Alpha band** |  |  |  |  |  |  |  |  |  |  |  |
| P-Word Retrieval  (*N* = 15) | *ᴛ* | - | - | - | - | - | - | - | - | - | - |
|  | *p* |  |  |  |  |  |  |  |  |  |  |
| P-Phonology  (*N* = 15) | *ᴛ* | - | - | - | - | - | - | - | - | - | - |
|  | *p* |  |  |  |  |  |  |  |  |  |  |
| P-Semantics  (*N* = 15) | *ᴛ* | - | - | - | - | - | - | - | - | - | - |
|  | *p* |  |  |  |  |  |  |  |  |  |  |
| P-Grammar  (*N* = 15) | *ᴛ* | - | - | - | 0.39 | - | - | - | - | - | - |
|  | *p* |  |  |  | .047 |  |  |  |  |  |  |
| C-Auditory Input  (*N* = 15) | *ᴛ* | - | - | - | - | - | - | - | - | - | - |
|  | *p* |  |  |  |  |  |  |  |  |  |  |
| C-Visual Input  (*N* = 13) | *ᴛ* | - | -0.50 | - | -0.60 | - | 0.44 | - | - | 0.44 | -0.46 |
|  | *p* |  | .022 |  | .005 |  | .040 |  |  | .046 | .034 |
| Reading  (*N* = 14) | *ᴛ* | - | - | - | - | - | - | - | - | - | - |
|  | *p* |  |  |  |  |  |  |  |  |  |  |
| Writing  (*N* = 13) | *ᴛ* | - | - | - | - | - | - | - | - | - | - |
|  | *p* |  |  |  |  |  |  |  |  |  |  |

*Note.* Only the tests with *p* < 0.05 are presented. *N* = group size; *ᴛ* = Kendall’s tau-b correlation coefficient; *p* = p-value (two-sided).; W = weighted: these network measures quantify weighted FC networks; MST = Minimum Spanning Tree: these network measures quantify Minimum Spanning Tree FC networks. FC = functional connectivity; PLI = Phase lag index, mean of all 16 remaining electrodes; rC = relative average clustering coefficient; rL = relative average path length; SWI = small-world index; MST-Degr = MST-maximum degree; MST-Ecc = MST-eccentricity, mean of all nodes; MST-BC = MST-maximum betweenness centrality; MST-Leaf = MST-leaf fraction; MST-Diam = MST-diameter; MST-TH = MST-tree hierarchy.
