# Supplementary material for: Resting-State Electroencephalography Functional Connectivity Networks Relate to Pre- and Postoperative Language Functioning in Low-Grade Glioma and Meningioma Patients
Source: Front Neurosci. 2021 Dec 8;15:785969. doi: 10.3389/fnins.2021.785969 (PMC8693574; doi:10.3389/fnins.2021.785969)
Supplement: Supplementary file 5 [file Table_5.docx]

**Appendix 5 – Glioma patients with vs. without language impairment**

*Theta- and alpha-band FC network characteristics of glioma patients with vs. without language impairment before surgery*

|  | Glioma patients with language impairment at T1  (*N* = 9) | | |  | Glioma patients without language impairment at T1  (*N* = 6) | | |  | Comparisons | |
| --- | --- | --- | --- | --- | --- | --- | --- | --- | --- | --- |
|  | *Mdn* | *Min* | *Max* |  | *Mdn* | *Min* | *Max* |  | *U* | *p* |
| **Theta band** |  |  |  |  |  |  |  |  |  |  |
| W-PLI | 0.135 | 0.114 | 0.207 |  | 0.121 | 0.107 | 0.146 |  | 15.0 | 0.181 |
| W-rC | 1.010 | 0.978 | 1.106 |  | 0.996 | 0.987 | 1.037 |  | 22.0 | 0.607 |
| W-rL | 0.901 | 0.885 | 0.955 |  | 0.902 | 0.884 | 0.908 |  | 23.0 | 0.689 |
| W-SWI | 1.121 | 1.089 | 1.163 |  | 1.104 | 1.097 | 1.173 |  | 27.0 | 1.000 |
| MST-Degr | 0.373 | 0.293 | 0.427 |  | 0.347 | 0.280 | 0.373 |  | 17.0 | 0.272 |
| MST-Ecc | 0.369 | 0.330 | 0.388 |  | 0.396 | 0.343 | 0.439 |  | 9.5 | *0.036* |
| MST-BC | 0.728 | 0.657 | 0.796 |  | 0.726 | 0.708 | 0.754 |  | 27.0 | 1.000 |
| MST-Leaf | 0.613 | 0.520 | 0.627 |  | 0.560 | 0.453 | 0.614 |  | 11.5 | 0.066 |
| MST-Diam | 0.453 | 0.400 | 0.480 |  | 0.494 | 0.427 | 0.547 |  | 6.0 | *0.012* |
| MST-TH | 0.422 | 0.379 | 0.439 |  | 0.385 | 0.311 | 0.417 |  | 9.0 | *0.036* |
| **Alpha band** |  |  |  |  |  |  |  |  |  |  |
| W-PLI | 0.166 | 0.108 | 0.245 |  | 0.139 | 0.103 | 0.197 |  | 19.0 | 0.388 |
| W-rC | 1.012 | 0.973 | 1.083 |  | 1.030 | 1.008 | 1.054 |  | 19.0 | 0.388 |
| W-rL | 0.911 | 0.887 | 0.929 |  | 0.905 | 0.888 | 0.957 |  | 18.0 | 0.328 |
| W-SWI | 1.106 | 1.077 | 1.170 |  | 1.122 | 1.093 | 1.188 |  | 17.0 | 0.272 |
| MST-Degr | 0.333 | 0.294 | 0.480 |  | 0.360 | 0.293 | 0.427 |  | 25.5 | 0.864 |
| MST-Ecc | 0.374 | 0.312 | 0.410 |  | 0.383 | 0.335 | 0.420 |  | 22.0 | 0.607 |
| MST-BC | 0.735 | 0.676 | 0.819 |  | 0.703 | 0.673 | 0.787 |  | 17.0 | 0.272 |
| MST-Leaf | 0.613 | 0.520 | 0.667 |  | 0.573 | 0.520 | 0.680 |  | 20.5 | 0.456 |
| MST-Diam | 0.467 | 0.387 | 0.520 |  | 0.473 | 0.413 | 0.520 |  | 24.0 | 0.776 |
| MST-TH | 0.407 | 0.378 | 0.429 |  | 0.388 | 0.381 | 0.475 |  | 25.0 | 0.864 |

*Note*. *Mdn* = median; *Min* = minimum value; *Max* = maximum value; *U* = test statistic of the Mann-Whitney U tests; *p* = p-value (two-sided). Comparisons with *p* < 0.05 are presented in italics.. W = weighted: these network measures quantify weighted FC networks; MST = Minimum Spanning Tree: these network measures quantify Minimum Spanning Tree FC networks. FC = functional connectivity; PLI = Phase lag index, mean of all 16 remaining electrodes; rC = relative average clustering coefficient; rL = relative average path length; SWI = small-world index; MST-Degr = MST-maximum degree; MST-Ecc = MST-eccentricity, mean of all nodes; MST-BC = MST-maximum betweenness centrality; MST-Leaf = MST-leaf fraction; MST-Diam = MST-diameter; MST-TH = MST-tree hierarchy.
