# Supplementary material for: Resting-State Electroencephalography Functional Connectivity Networks Relate to Pre- and Postoperative Language Functioning in Low-Grade Glioma and Meningioma Patients
Source: Front Neurosci. 2021 Dec 8;15:785969. doi: 10.3389/fnins.2021.785969 (PMC8693574; doi:10.3389/fnins.2021.785969)
Supplement: Supplementary file 6 [file Table_6.docx]

**Appendix 6 – Meningioma patients with vs. without language impairment**

*Theta- and alpha-band FC network characteristics of meningioma patients with vs. without language impairment before surgery*

|  | Meningioma patients with language impairment at T1  (*N* = 4) | | |  | Meningioma patients without language impairment at T1  (*N* = 6) | | |  | Comparisons | |
| --- | --- | --- | --- | --- | --- | --- | --- | --- | --- | --- |
|  | *Mdn* | *Min* | *Max* |  | *Mdn* | *Min* | *Max* |  | *U* | *p* |
| **Theta band** |  |  |  |  |  |  |  |  |  |  |
| W-PLI | 0.134 | 0.124 | 0.138 |  | 0.131 | 0.113 | 0.449 |  | 12.0 | 1.000 |
| W-rC | 0.991 | 0.966 | 1.038 |  | 1.013 | 0.952 | 1.061 |  | 10.0 | 0.762 |
| W-rL | 0.896 | 0.888 | 0.898 |  | 0.915 | 0.896 | 1.012 |  | 2.0 | *0.038* |
| W-SWI | 1.110 | 1.079 | 1.156 |  | 1.092 | 1.051 | 1.122 |  | 7.0 | 0.352 |
| MST-Degr | 0.347 | 0.320 | 0.386 |  | 0.327 | 0.267 | 0.547 |  | 8.0 | 0.476 |
| MST-Ecc | 0.375 | 0.339 | 0.405 |  | 0.388 | 0.296 | 0.429 |  | 10.0 | 0.762 |
| MST-BC | 0.729 | 0.707 | 0.737 |  | 0.717 | 0.707 | 0.874 |  | 11.5 | 0.914 |
| MST-Leaf | 0.567 | 0.520 | 0.640 |  | 0.527 | 0.453 | 0.653 |  | 8.0 | 0.476 |
| MST-Diam | 0.467 | 0.413 | 0.507 |  | 0.487 | 0.360 | 0.547 |  | 9.0 | 0.610 |
| MST-TH | 0.393 | 0.367 | 0.435 |  | 0.361 | 0.321 | 0.409 |  | 6.0 | 0.257 |
| **Alpha band** |  |  |  |  |  |  |  |  |  |  |
| W-PLI | 0.143 | 0.110 | 0.211 |  | 0.212 | 0.107 | 0.274 |  | 8.0 | 0.476 |
| W-rC | 1.016 | 0.976 | 1.034 |  | 1.012 | 0.980 | 1.068 |  | 11.0 | 0.914 |
| W-rL | 0.902 | 0.894 | 0.911 |  | 0.916 | 0.889 | 0.944 |  | 7.0 | 0.352 |
| W-SWI | 1.125 | 1.093 | 1.140 |  | 1.121 | 1.045 | 1.160 |  | 11.0 | 0.914 |
| MST-Degr | 0.373 | 0.307 | 0.493 |  | 0.347 | 0.280 | 0.427 |  | 7.5 | 0.352 |
| MST-Ecc | 0.373 | 0.311 | 0.418 |  | 0.361 | 0.325 | 0.459 |  | 12.0 | 1.000 |
| MST-BC | 0.768 | 0.670 | 0.798 |  | 0.732 | 0.686 | 0.751 |  | 7.5 | 0.352 |
| MST-Leaf | 0.587 | 0.560 | 0.693 |  | 0.580 | 0.480 | 0.667 |  | 8.5 | 0.476 |
| MST-Diam | 0.467 | 0.373 | 0.520 |  | 0.447 | 0.400 | 0.587 |  | 12.0 | 1.000 |
| MST-TH | 0.405 | 0.378 | 0.437 |  | 0.392 | 0.350 | 0.464 |  | 10.0 | 0.762 |

*Note*. *Mdn* = median; *Min* = minimum value; *Max* = maximum value; *U* = test statistic of the Mann-Whitney U tests; *p* = p-value (two-sided). Comparisons with *p* < 0.05 are presented in italics. W = weighted: these network measures quantify weighted FC networks; MST = Minimum Spanning Tree: these network measures quantify Minimum Spanning Tree FC networks. FC = functional connectivity; PLI = Phase lag index, mean of all 16 remaining electrodes; rC = relative average clustering coefficient; rL = relative average path length; SWI = small-world index; MST-Degr = MST-maximum degree; MST-Ecc = MST-eccentricity, mean of all nodes; MST-BC = MST-maximum betweenness centrality; MST-Leaf = MST-leaf fraction; MST-Diam = MST-diameter; MST-TH = MST-tree hierarchy.
