# Supplementary material for: Resting-State Electroencephalography Functional Connectivity Networks Relate to Pre- and Postoperative Language Functioning in Low-Grade Glioma and Meningioma Patients
Source: Front Neurosci. 2021 Dec 8;15:785969. doi: 10.3389/fnins.2021.785969 (PMC8693574; doi:10.3389/fnins.2021.785969)
Supplement: Supplementary file 7 [file Table_7.DOCX]

**Appendix 7 – FC network characteristics at T1 and language outcome at T2 in glioma patients**

*Correlation analyses between FC network measures at T1 and language outcome (domain z-scores) at T2 in glioma patients*

|  |  | W-PLI | W-  rC | W-  rL | W-SWI | MST-Degr | MST-Ecc | MST-BC | MST-Leaf | MST-Diam | MST-TH |
| --- | --- | --- | --- | --- | --- | --- | --- | --- | --- | --- | --- |
| **Theta band** |  |  |  |  |  |  |  |  |  |  |  |
| P-Word Retrieval  (*N* = 13) | *ᴛ* | -0.57 | -0.44 | - | - | -0.49 | 0.69 | - | -0.57 | 0.73 | -0.49 |
|  | *p* | .008 | .042 |  |  | .022 | .001 |  | .009 | .001 | .023 |
| P-Phonology  (*N* = 13) | *ᴛ* | - | - | - | - | - | - | - | - | - | - |
|  | *p* |  |  |  |  |  |  |  |  |  |  |
| P-Semantics  (*N* = 13) | *ᴛ* | - | - | - | - | - | - | - | - | - | - |
|  | *p* |  |  |  |  |  |  |  |  |  |  |
| P-Morphosyntax  (*N* = 13) | *ᴛ* | - | - | - | - | - | - | - | - | - | - |
|  | *p* |  |  |  |  |  |  |  |  |  |  |
| C-Auditory Input  (*N* = 13) | *ᴛ* | - | - | - | - | - | - | - | - | - | - |
|  | *p* |  |  |  |  |  |  |  |  |  |  |
| C-Visual Input  (*N* = 12) | *ᴛ* | - | - | - | - | - | - | - | - | - | - |
|  | *p* |  |  |  |  |  |  |  |  |  |  |
| Reading  (*N* = 13) | *ᴛ* | - | - | - | - | - | - | - | - | - | - |
|  | *p* |  |  |  |  |  |  |  |  |  |  |
| Writing  (*N* = 13) | *ᴛ* | -0.47 | - | - | - | - | - | - | - | - | - |
|  | *p* | .036 |  |  |  |  |  |  |  |  |  |
| **Alpha band** |  |  |  |  |  |  |  |  |  |  |  |
| P-Word Retrieval  (*N* = 13) | *ᴛ* | - | - | - | - | - | - | - | - | - | - |
|  | *p* |  |  |  |  |  |  |  |  |  |  |
| P-Phonology  (*N* = 13) | *ᴛ* | - | - | - | - | - | - | - | - | - | - |
|  | *p* |  |  |  |  |  |  |  |  |  |  |
| P-Semantics  (*N* = 13) | *ᴛ* | - | - | - | - | - | - | - | - | - | - |
|  | *p* |  |  |  |  |  |  |  |  |  |  |
| P-Morphosyntax  (*N* = 13) | *ᴛ* | - | - | - | - | - | - | - | - | - | - |
|  | *p* |  |  |  |  |  |  |  |  |  |  |
| C-Auditory Input  (*N* = 13) | *ᴛ* | - | - | - | - | - | - | - | - | - | - |
|  | *p* |  |  |  |  |  |  |  |  |  |  |
| C-Visual Input  (*N* = 12) | *ᴛ* | - | - | - | - | - | - | - | - | - | - |
|  | *p* |  |  |  |  |  |  |  |  |  |  |
| Reading  (*N* = 13) | *ᴛ* | - | - | - | - | - | - | - | - | - | - |
|  | *p* |  |  |  |  |  |  |  |  |  |  |
| Writing  (*N* = 13) | *ᴛ* | - | - | - | - | - | - | - | - | - | - |
|  | *p* |  |  |  |  |  |  |  |  |  |  |

*Note.* Only the tests with *p* < 0.05 are presented. *N* = group size; *ᴛ* = Kendall’s tau-b correlation coefficient; *p* = p-value (two-sided).. P = production; C = comprehension; W = weighted: these network measures quantify weighted FC networks; MST = Minimum Spanning Tree: these network measures quantify Minimum Spanning Tree FC networks. FC = functional connectivity; PLI = Phase lag index, mean of all 16 remaining electrodes; rC = relative average clustering coefficient; rL = relative average path length; SWI = small-world index; MST-Degr = MST-maximum degree; MST-Ecc = MST-eccentricity, mean of all nodes; MST-BC = MST-maximum betweenness centrality; MST-Leaf = MST-leaf fraction; MST-Diam = MST-diameter; MST-TH = MST-tree hierarchy.
